# Gender differences in psychiatric outpatients: a before and during COVID-19 pandemic study in general hospitals from China

**DOI:** 10.1186/s12991-022-00412-3

**Published:** 2022-09-07

**Authors:** Wenli He, Danhong Xu, Jiafeng Wang, Yuze Shen, Zheng Lin, Liemin Ruan, Qiaozhen Chen

**Affiliations:** 1 Department of Psychiatry, the Second Affiliated Hospital, Zhejiang University School of Medicine, 88 Jiefang Road, Hangzhou, 310009 Zhejiang China; 2 Department of Psychiatry, the Second People’s Hospital of Yuhuan, Taizhou, China; 3 Department of Psychiatry, the First People’s Hospital of Yuhang District, Hangzhou, China; 4 Department of Psychosomatic Medicine, Ningbo First Hospital, Ningbo Hospital of Zhejiang University, No. 59 Liuting Street, Ningbo, 315010 Zhejiang China

**Keywords:** Mental disorder, General hospital, Gender, COVID-19, Outpatient

## Abstract

**Background:**

Little is known about the gender characteristics and the Corona Virus Disease 2019 (COVID-19) impact on psychiatric department outpatients in general hospitals in China.

**Methods:**

We retrospectively collected 225,947 outpatient clinic records before and during COVID-19 pandemic from January 1, 2019 to December 31, 2020 in the psychiatric clinic of 3 general hospitals, gender composition of patients was analyzed in different five age groups and nine diagnostic categories at three levels: total patient visits, number of patients and number of first-visit patients.

**Results:**

The total male-to-female ratio of psychiatric outpatient records in 3 general hospitals from 2019 to 2020 was 1:1.69. Women were more common in all age groups. Overall, the proportion of women was further increased in 2020 compared to that in 2019, especially in age group below 34 years and anxiety disorders category. Most mental disorders showed higher demands for females than that for males except mental and behavioral disorders due to psychoactive substance use with the male-to-female ratio was 1:0.05.

**Conclusions:**

The demand for female psychiatric outpatient services is obviously higher than that for males. It is necessary to pay more attention to explore targeted mechanism or psychosocial service strategy for female patients with mental disorders.

*Trial registration* ChiCTR2100044894, March 31, 2021 retrospectively registered.

**Supplementary Information:**

The online version contains supplementary material available at 10.1186/s12991-022-00412-3.

## Background

Mental disorders are getting more and more common in daily life. According to the latest epidemiological survey in China, the lifetime prevalence of mental disorders in 2013 was 16.6%, which was significantly higher than that in 2002(13.2%)[1]. The global burden of disease study in 2017 [2] showed that the global burden of mental disorders increased 31.6% from 1990 to 2007 and 13.5% from 2007 to 2017. It is well-known that there are gender differences in some specific mental disorders, for example, female patients have a higher prevalence in mood disorders and anxiety disorders, while mental and behavioral disorders due to psychoactive substance use are more common in male patients [1, 3].

However, the use of mental health service is different by gender. Previous studies [4] have investigated the distribution of gender in outpatients or emergency patients with mental disorders. In the 11- to 24-year-old population who were hospitalized in emergency department due to substance use and mental disorders, the proportion of men and women was 45.9% and 54.1%, respectively, in 1997–2010. From 2014 to 2015, 17.36% male adults and 28.27% female adults received mental health services in the United States [5]. Women were found to be more likely to seek mental health service. Most previous outpatient surveys describing gender proportion of psychiatric disorders in China were concentrated on investigating the prevalence of mental disorders in non-psychiatric departments [6, 7]. However, there is still a lack of research that can directly reflect the actual clinical needs of different genders in psychiatric outpatients of general hospitals in China.

In December 2019, COVID-19 broke out in Wuhan, China, and quickly became a pandemic all over the world. As of June 10, 2021, World Health Organization has reported 173,989,093 confirmed cases and 3,756,947 deaths of COVID-19 [8]. This public health emergency not only threatens people's health, but also has a great impact on their mental health. Previous studies reported that the pandemic of COVID-19 resulted in high rates of anxiety, depression and stress symptoms in the general population as well as in healthcare workers [9, 10]. Moreover, a preliminary study conducted in a general hospital in Chengdu, China, found that 20.9% of outpatients with history of mental disorders reported exacerbated symptoms due to pandemic of COVID-19 [11]. Therefore, it is important to look into the impact of the epidemic on psychiatric outpatient visits in general hospitals.

So far, we have not found any research describing the gender differences and the possible COVID-19 effect for psychiatric outpatients in general hospitals in China especially. Therefore, this survey was carried out, aiming to obtain the corresponding data support to understand the actual needs by gender in general hospitals and the influence of COVID-19 pandemic on male and female psychiatric outpatient visits, so as to provide the basis for the policy-making of relevant departments and the allocation of medical resources in the future.

## Methods

### Participants and data sources

The data of this study were collected from the psychiatric outpatient records of 3 general hospitals in Zhejiang province, China, including the Second Affiliated Hospital Zhejiang University School of Medicine, the Second People's Hospital of Yuhuan, and the First People's Hospital of Yuhang District, which were representative provincial, municipal and district general hospitals, respectively. This study was approved by the Human research ethics committee of the above three hospitals.

The subjects of this study were patients who visited the psychiatric clinics of above three general hospitals from January 1, 2019 to December 31, 2020. The inclusion criteria were the records with the diagnosis in accordance with the *Mental and behavioral disorders* of the International Statistical Classification of Diseases and Related Health Problems—10th edition (ICD-10) [12]. The exclusion criteria were the records with diagnosis that did not meet the ICD-10 *Mental and behavioral disorders*, the records of repeated visits within the same day, and the records with incomplete information and errors.

### Measures

Using Hospital Information System, outpatient clinic records were collected from January 1, 2019 to December 31, 2020 in the psychiatric department, including unique number, gender, age and diagnoses. The records of diagnosis not in accordance with ICD-10 *Mental and behavioral disorders* and the records of repeated visits within the same day, incomplete information and errors were excluded. Then, the age stratification and disease spectrum were reclassified. Age was divided into five groups including 0–18 years, 19–34 years, 35–49 year, 50–64 years and 65 years and over. We used the definite diagnosis as the diagnosis for all the records when there were several visiting records of a unique number. The main diagnosis was selected according to the diagnostic principle of mental disorder hierarchy, ranked from high level to low level were organic, psychotic, affective, neurotic, personality disorder and maladjustment. The high-level diagnosis was selected when there were multiple diagnoses. Diagnoses were classified according to the corresponding ICD code in the Hospital Information System. Then the diagnoses were categorized into nine groups consisting of dementia, mental and behavioral disorders due to psychoactive substance use, schizophrenia and other psychotic disorders, mood disorders, anxiety disorders, eating disorders, sleep disorders, other mental disorders and undetermined diagnoses. Classification principles were based on the ICD-10 *Mental and behavioral disorders* (Additional file 1: Table S1). Dementia contained dementia in Alzheimer disease, vascular dementia, dementia in other diseases classified elsewhere and unspecified dementia. Schizophrenia and other psychotic disorders included all diagnoses under the ICD-10 section of *Schizophrenia, schizotypal and delusional disorders*. Mood disorder included all diagnoses under the ICD-10 section of *Mood [affective] disorders*. Anxiety disorders included all diagnoses under the ICD-10 section of *Neurotic, stress-related and somatoform disorders*. Other mental disorders included all diagnoses under the ICD-10 section of *Organic, including symptomatic, mental disorders*(except dementia), *Behavioral syndromes associated with physiological disturbances and physical factors*(except eating disorders and sleep disorders), *Disorders of adult personality and behavior*, *Mental retardation*, *Disorders of psychological development*, *Behavioral and emotional disorders with onset usually occurring in childhood and adolescence* and *Unspecified mental disorder*. Undetermined diagnoses included mental disorders to be determined (such as schizophrenia to be determined, dementia to be determined, etc.), symptom description (such as hallucination, agitation, anxiety state, depression state, etc.) and psychotherapy (with no definite diagnosis of mental illness before and after treatment). The total patient visits per year were defined as the total number of the reclassified visit records. The number of patients per year was defined as the person-time of all different unique number. The number of first-visit patients was defined as the person-time of all different unique number which did not appear in the previous year.

### Statistical analysis

Data were analyzed using SPSS25.0 (SPSS Inc., Chicago, USA). Non-normally distributed measurement data were described by median and 95% confidence interval. The enumeration data were described by constituent ratio, and Chi-square test was used to compare the differences between groups or years. *P* < 0.05 was set as the difference with statistical significance, and *P* < 0.001 as extremely significant statistical difference.

## Results

**Fig.1:**
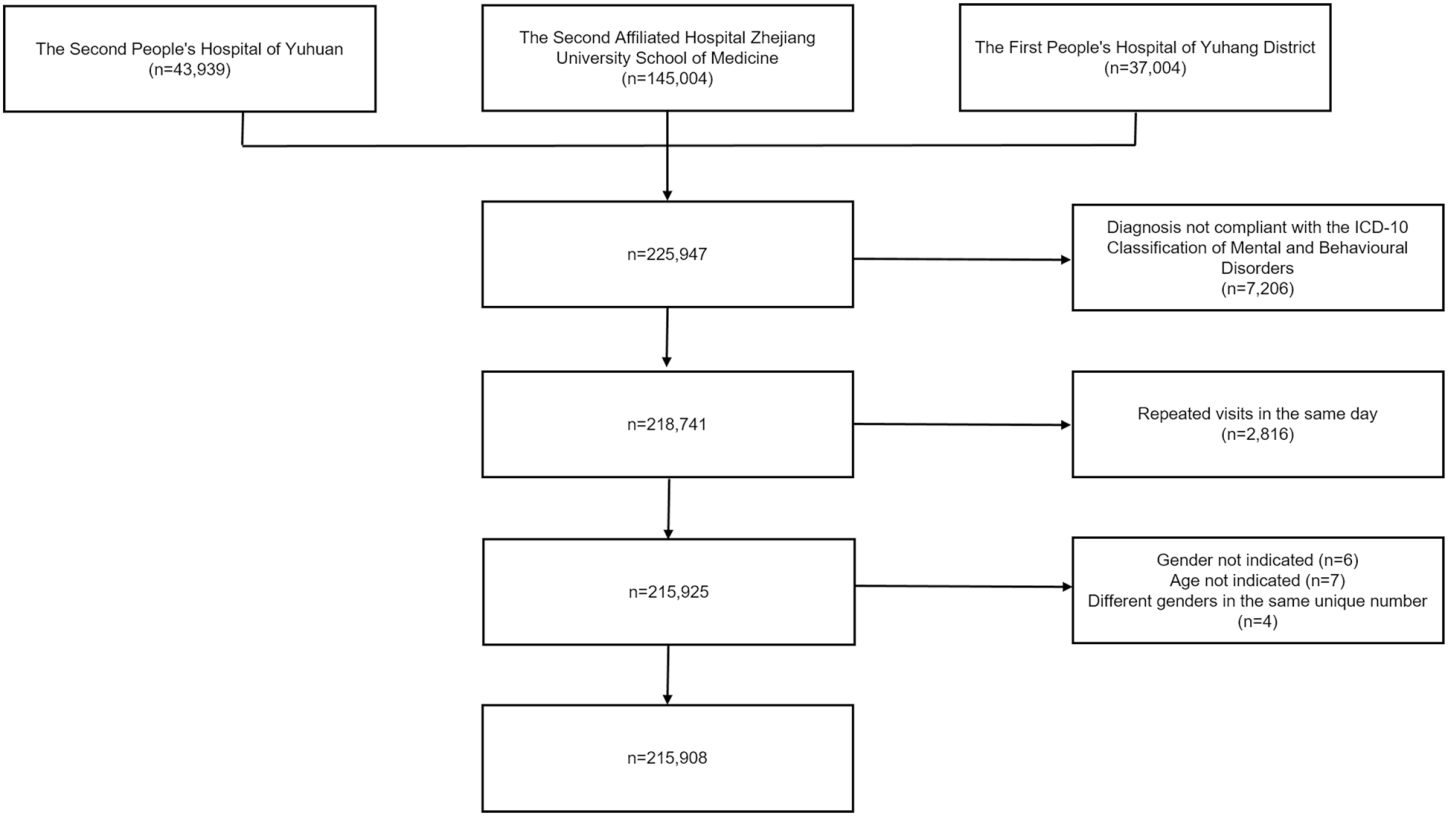
Flowchart of data filtering

As shown in Fig. 1, we identified a total of 225,947 person-time visiting records between 2019 and 2020, collecting information such as gender, age, unique number, diagnosis and visiting date. After removing unqualified records, 215,908 records were finally included and then sorted out by age groups and diagnosis categories.

**Fig.2:**
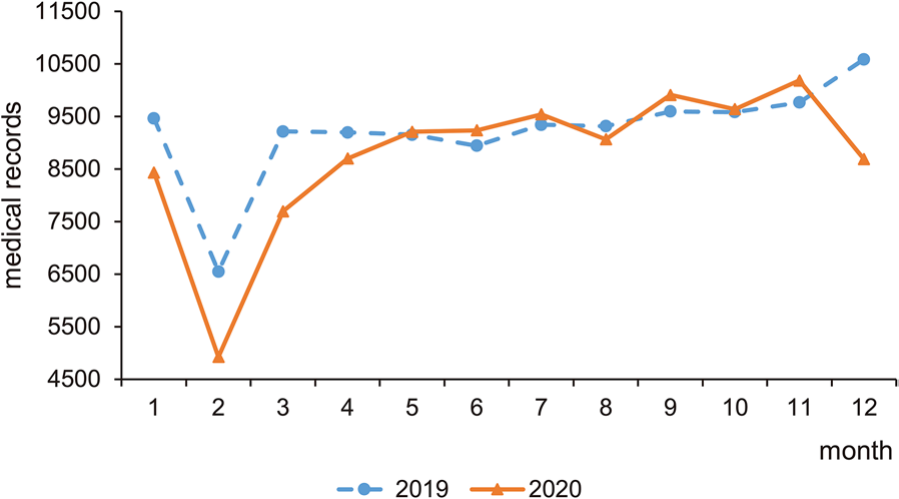
Monthly distribution of medical records of psychiatric outpatients in 2019 and 2020

In the total medical records of the 3 hospitals from 2019 to 2020, age ranged from 3 to 103 years, with a median age of 44 [14–79] years, and the male-to-female ratio was 1:1.69. The median visit frequency of male and female patients was 2 [1–20] and 2 [1–19], respectively. Anxiety disorder, mood disorder, undetermined diagnoses, sleep disorders, schizophrenia and other psychotic disorders were the most common diagnostic categories, accounting for 36.83%, 16.60%, 15.45%, 13.57% and 9.40%, respectively (see Table 1). The number of medical records in January, February, March, April and December was decreased in 2020 compared to that in 2019 (see Fig. 2).

**Table 1:** Characteristics of total medical records of psychiatric outpatients from 2019 to 2020(*n*=215,908)

	*n*	%
Age group		
0-18	23,296	10.79
19-34	52,145	24.15
35-49	54,357	25.18
50-64	55,917	25.90
≥65	30,193	13.98
Gender		
Male	80,356	37.22
Female	135,552	62.78
Diagnostic category		
Dementia	364	0.17
Mental disorders due to psychoactive substance use	796	0.37
Schizophrenia and other psychotic disorders	20,303	9.40
Mood disorders	35,842	16.60
Anxiety disorders	79,521	36.83
Eating disorders	615	0.28
Sleep disorders	29,305	13.57
Other mental disorders	15,804	7.32
Undetermined diagnoses	33,358	15.45
Year		
2019	110,699	51.27
2020	105,209	48.73
Data sources		
The Second Affiliated Hospital Zhejiang University School of Medicine	139,324	64.53
The Second People's Hospital of Yuhuan	41,160	19.06
The First People's Hospital of Yuhang District	35,424	16.41

### Gender difference in years

**Fig.3:**
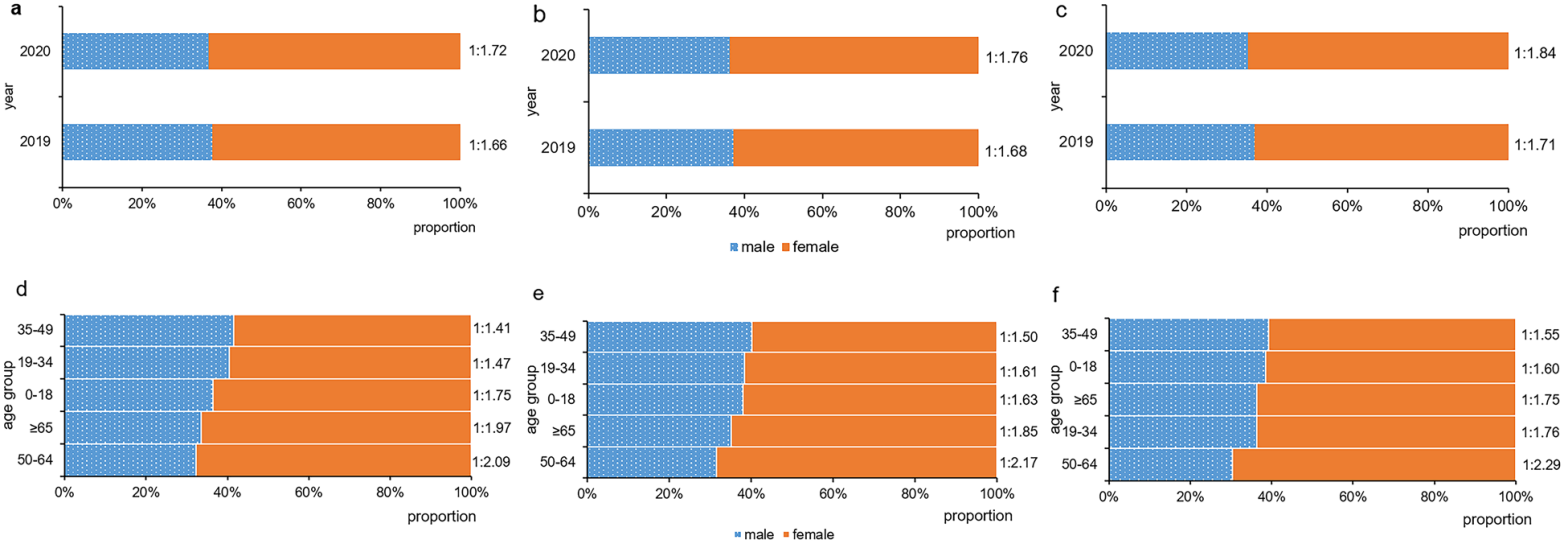
The gender differences in years and age groups. The figure represents the gender composition in 2019 and 2020 at the level of total patient visits (**a**), number of patients (**b**) and number of first-visit patients (**c**), and by age groups in 2019–2020 at the level of total patient visits (**d**), number of patients (**e**) and number of first-visit patients (**f**)

Figure 3 and Additional file 1: Table S2–S4 show a significantly increased proportion of female psychiatric outpatients in 2020 at the level of total patient visits (*χ*^*2*^ = 18.308, *P* < 0.001), patient number (*χ*^*2*^ = 8.736, *P* = 0.003) and first-visit patient number (*χ*^*2*^ = 12.693, *P* < 0.001).

### Gender differences in 5 age groups

At the 3 levels, female patients were more common in all age groups in psychiatric clinic, with 50–64 years group owned the greatest difference and 35–49 years group the least. Interestingly, the female proportion of 19–34 years group ranked the second at the level of first-visit patient number, while 65 years and over group was the second at other 2 levels (see Fig. 3 and Additional file 1: Table S2–S4).

**Fig.4:**
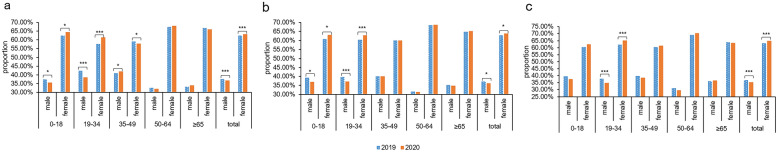
The gender composition change of different age groups in 2019–2020. The figure represents the change of gender composition in different age groups from 2019 to 2020 at the level of total patient visits (**a**), number of patients (**b**) and number of first-visit patients (**c**). *: *p* < 0.05. ***: *p* < 0.001

The female proportion of group aged 0–18 and 19–34 years in 2020 was significantly increased when compared to that of 2019, especially in 19–34 years group at the level of total patient visits (*χ*^*2*^ = 71.657, *P* < 0.001), patient number (*χ*^*2*^ = 13.895, *P* < 0.001) and first-visit patient number (*χ*^*2*^ = 13.603, *P* < 0.001) (see Fig. 4, Additional file 1: Table S5).

### Gender differences in 9 diagnostic categories

**Fig.5:**
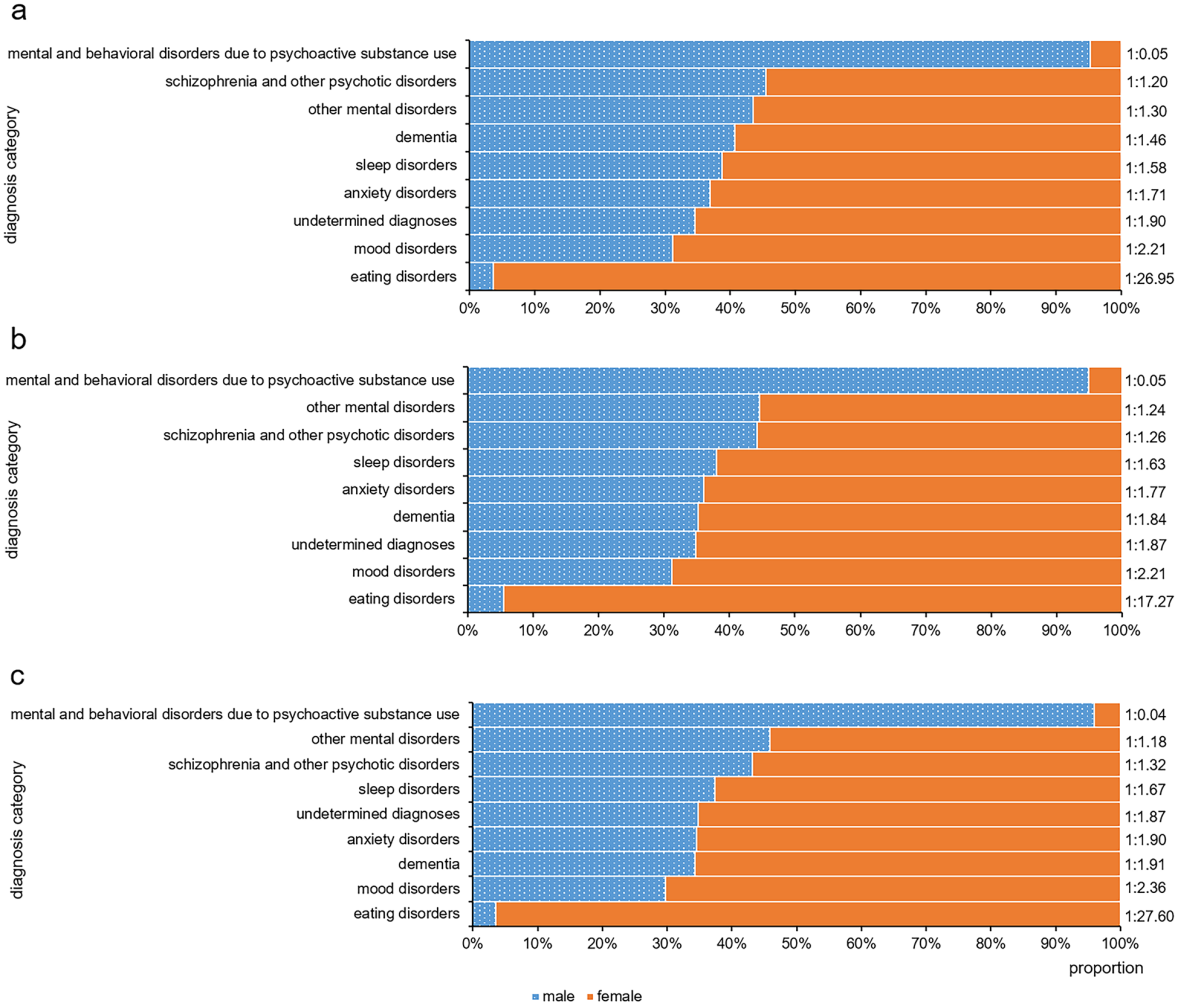
The gender differences in diagnostic categories. The figure represents the gender composition by diagnostic categories in 2019–2020 at the level of total patient visits (**a**), number of patients (**b**) and number of first-visit patients (**c**)

At the three levels, female patients were more common in all diagnosis categories except mental and behavioral disorders due to psychoactive substance use, as shown in Fig. 5. Eating disorder and mood disorder were the first two categories with the highest proportion of women.

**Fig.6:**
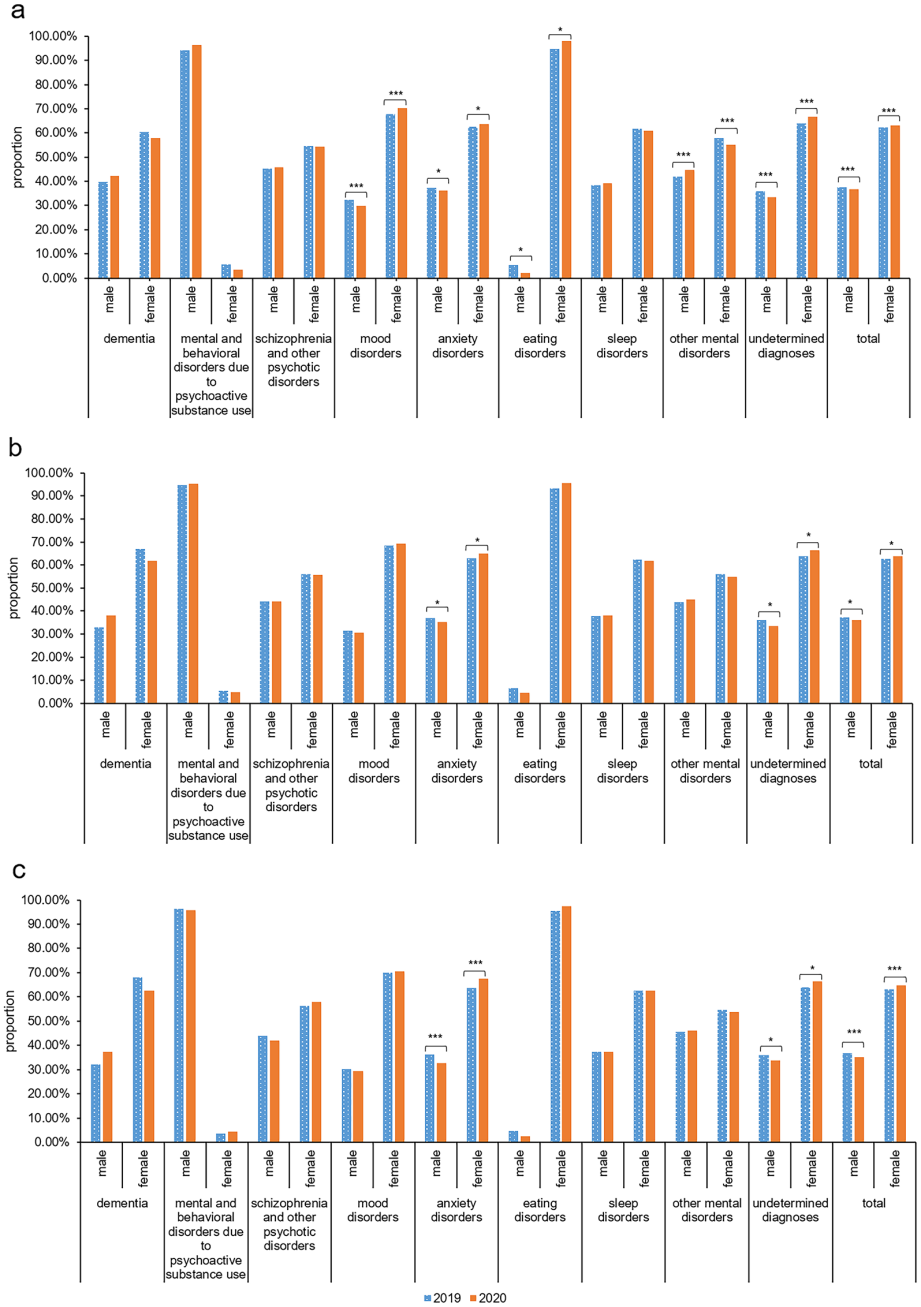
The gender composition change of different diagnostic categories in 2019–2020. The figure represents the change of gender composition in different diagnostic categories from 2019 to 2020 at the level of total patient visits (**a**), number of patients (**b**) and number of first-visit patients (**c**). *: *p* < 0.05. ***: *p* < 0.001

The female proportion of anxiety disorder category in 2020 was significantly increased at the level of total patient visits (*χ*^*2*^ = 9.781, *P* < 0.05), patient number (*χ*^*2*^ = 7.801, *P* < 0.05) and first-visit patient number (*χ*^*2*^ = 15.655, *P* < 0.001) when compared to that of 2019 (see Fig. 6, Additional file 1: Table S6). The female proportion of undetermined diagnoses category in 2020 was significantly increased at the level of total patient visits (*χ*^*2*^ = 27.142, *P* < 0.001), patient number (*χ*^*2*^ = 11.041, *P* < 0.05) and first-visit patient number (*χ*^*2*^ = 8.458, *P* < 0.05) when compared to that of 2019 (see Fig. 6, Additional file 1: Table S6).

## Discussion

To the best of our knowledge, this is the first full-sample data study that describes the gender distribution of psychiatric outpatients in three general hospitals in China, which provides data support for the needs of different genders in psychiatric clinical practice. In this study, women were more common in psychiatric clinics in general hospitals, and the difference in the male-to-female ratio had increased. Besides, women are more common in all age groups, especially in the group aged 50–64 years, as well as in almost of diagnostic categories except the category of mental and behavioral disorders due to psychoactive substance use. Patients with 19–34 years group and anxiety disorders, undetermined diagnosis categories showed an increased proportion of women.

Women suffering from mental disorders were more common in psychiatric clinics in general hospitals. We found an increased proportion in women from 2019 to 2020 at the level of total patient visits, number of patients and number of first-visit patients. The results were obviously contrary to the census data in China, which reported that there were more males than females and the male-to-female ratio reached 1.05:1 [13]. The constituent ratio of psychiatric clinic visits in females in this study was significantly higher than that of males, a converse result with the population census, which might be related to the diagnosis of mild and affective diseases predominating in psychiatric clinics of general hospitals, such as anxiety disorders and mood disorders, in which women had a higher prevalence than men [14–16]. In addition, a study in Australia found that men sought mental health services less frequently than women, with 5.33% of men and 10.2% of women receiving mental health services [17]. A study conducted in the Netherlands found that female patients with anxiety disorders in psychiatric clinics had higher scores on the self-rating scale than men [18]. The prevalence of mental disorders and the burden of disease have increased, while women feel worse about themselves and have more desire for treatment and expression, which may be related to the obvious gender differences in general hospitals. Furthermore, the negative effects of COVID-19 might exacerbate the risk of mental disorders in women and expand the gender difference, such as increased domestic violence against women [19], heavier economic burden, overuse of Internet and social media [20], which were the risk factors of mental illness. The significant gender gap indicates the great demand for women's diagnosis and treatment, which prompted the government and relevant departments and organizations to take targeted measures, especially to strengthen the care and attention to women's mental health. Besides, some male patients with mental illness who have not sought medical treatment also need to be concerned.

The gender composition of all age groups was dominated by women, and the gender difference in the 50–64 age group was the highest at the level of person-time of diagnosis and treatment, patient number and first-visit patient number, followed by group aged 65 years and over at the level of total patient visits and patient numbers. However, at the level of first-visit patient number, the gender difference in the 19–34 age group ranked second among all age groups. Besides, 0–18 and 19–34 age group showed a significantly increased female proportion in 2020. Our results corresponded with a previous study from Austria, which reported that people under 35 years and female sex showed more mental health symptoms during COVID-19 [21]. The widening of the gender gap in adolescents and young adults might be related to the following reasons. Firstly, adolescents and young adults use social media more frequently than the old [20]. It means that they may be exposed to more information of the pandemic and panic. Meanwhile, women are more prone to worse psychological changes than men [22]. Secondly, older people may experience more life events than young and have stronger psychological resilience [23]. Previous research reported that older adults had lower perceived stress than younger one [24]. Our results indicated that more attention and support should be paid to adolescent and young adult women and more specific measures should be developed to improve this situation.

The proportion of male patients with mental and behavioral disorders due to psychoactive substance use was significantly higher than that of female patients, but in the other diagnostic categories the proportion of female patients was higher than males. This result was basically consistent with the gender distribution in the previous epidemiological surveys of the prevalence of mental disorders [25]. The female proportion of anxiety disorders and undetermined diagnosis categories was significantly increased in 2020 at all three levels. Several previous studies had illustrated high rates of anxiety and depression symptoms during COVID-19 [9, 26]. But in this study, it was interesting to find that female proportion in mood disorders did not show similar trend at all three levels. This finding still needs further studies to verify. Increased female proportion of undetermined diagnosis categories partly reflected worse mental health condition in female and it was consistent with the result that the proportion of first-visit female patients were increased. As there are a large number of diagnoses in the categories of other mental disorders and undetermined diagnosis, further stratified analysis and longitudinal follow-up can be carried out to explore the development of diseases with undetermined diagnosis.

Our study has several limitations. First, this is a retrospective study, which lacks comprehensive longitudinal follow-up investigation. Due to the large number of outpatients and the limited time for each patient to receive diagnosis and treatment, it is possible that the outpatient diagnosis records involved are not completely accurate. Although electronic medical records have been popularized in recent years, the diagnosis basis of each hospital is not completely consistent and homogeneous, so there may be some errors in the classification of diagnosis. In addition, this study used monism, did not analyze the comorbidity of mental disorders, there may be some errors in the gender distribution of each diagnosis. And this study only described and compared the diagnostic categories, but did not further analyze the diagnostic subclasses. More targeted data analysis can be carried out in the future.

## Conclusions

This study found that women have significantly higher demand for psychiatric clinics in general hospital. The proportion of women was further increased in 2020 compared to that of 2019, especially in adolescent and young adult group and anxiety disorders category which might be partly affected by COVID-19. Female outpatients were more common in all age groups and more common in most diagnosis categories except mental and behavioral disorders due to psychoactive substance use. This provided data support for the current needs of patients in the psychiatric clinic of general hospitals in China. Therefore, it is necessary to further explore the potential mechanisms in gender and strengthen attention to women’s mental health prevention and treatment.

## Supplementary Information

**Additional file 1:** **Table S1.** Diagnostic classification. **Table S2.** Gender composition of age, diagnosis and year at the level of person-time of diagnosis and treatment. **Table S3.** Gender composition of age, diagnosis and year at the level of patient number. **Table S4.** Gender composition of age, diagnosis and year at the level of first-visit patient number. **Table S5.** Gender difference by age groups in 2019 and 2020. **Table S6.** Gender difference by diagnostic categories in 2019 and 2020

## Data Availability

The data in this study are not publicly available due to data protection.
