# Supplementary material for: Gender differences in psychiatric outpatients: a before and during COVID-19 pandemic study in general hospitals from China
Source: Ann Gen Psychiatry. 2022 Sep 7;21:35. doi: 10.1186/s12991-022-00412-3 (PMC9450837; doi:10.1186/s12991-022-00412-3)
Supplement: Supplementary file 1 — Additional file 1: Table S1. Diagnostic classification. Table S2. Gender composition of age, diagnosis and year at the level of person-time of diagnosis and treatment. Table S3. Gender composition of age, diagnosis and year at the level of patient number. Table S4. Gender composition of age, diagnosis and year at the level of first-visit patient number. Table S5. Gender difference by age groups in 2019 and 2020. Table S6. Gender difference by diagnostic categories in 2019 and 2020 [file 12991_2022_412_MOESM1_ESM.pdf]

Online Resource

Gender differences in psychiatric outpatients: a before and during COVID-19 pandemic study in general hospitals from China

European Archives of Psychiatry and Clinical Neuroscience

Wenli He[1], Danhong Xu[1,2], Jiafeng Wang[1], Yuze Shen[1,3], Zheng Lin[1], Liemin Ruan[4,*], Qiaozhen Chen[1,*]

1 Department of Psychiatry, the Second Affiliated Hospital, Zhejiang University School of Medicine, Hangzhou, China

2 Department of Psychiatry, the Second People's Hospital of Yuhuan, Taizhou, China

3 Department of Psychiatry, the First People's Hospital of Yuhang District, Hangzhou, China

4 Department of Psychosomatic Medicine, Ningbo First Hospital, Ningbo, China

*Correspondence author: Dr. Qiaozhen Chen

Department of Psychiatry, the Second Affiliated Hospital,

Zhejiang University School of Medicine

88 Jiefang Road, Hangzhou, Zhejiang 310009, China

E-mail address: crazil@zju.edu.cn

*Co-corresponding author: Dr. Liemin Ruan

Department of Psychosomatic Medicine, Ningbo First Hospital,

Ningbo Hospital of Zhejiang University

No. 59 Liuting Street, Ningbo, Zhejiang 315010, China

E-mail address: lmruan@tom.com

Table S1 Diagnostic classification

ICD-10 code	Diagnostic categories	Clinical diagnosis
F00-F03	Dementia	Dementia
		Dementia in Alzheimer disease
		Vascular dementia
		Dementia in Parkinson disease
		Neurosyphilis
F10-F19	Mental and behavioral disorders due to psychoactive substance use	Mental and behavioral disorders due to use of alcohol
		Mental and behavioral disorders due to use of tobacco
		Mental and behavioral disorders due to multiple drug use and use of other psychoactive substances
		Mental and behavioral disorders due to psychoactive substance use
F20-F29	Schizophrenia and other psychotic disorders	Schizophrenia
		Schizotypal disorder
		Delusional disorder
		Acute and transient psychotic disorders
		Schizoaffective disorders
		Paranoid disorder
F30-F39	Mood disorders	Mood [affective] disorders
		Hypomania
		Manic episode
		Bipolar affective disorder
		Depressive episode
		Recurrent depressive disorder
		Cyclothymia
		Dysthymia
		Mixed affective episode
F40-F48	Anxiety disorders	Phobic anxiety disorders
		Panic disorder [episodic paroxysmal anxiety]
		Generalized anxiety disorder
		Mixed anxiety and depressive disorder
		Anxiety disorders
		Obsessive-compulsive disorder
		Acute stress reaction
		Post-traumatic stress disorder
		Adjustment disorders

ICD-10 code	Diagnostic categories	Clinical diagnosis
		Dissociative [conversion] disorders
		Somatoform disorders
		Neurasthenia
F50	Eating disorders	Anorexia nervosa
		Bulimia nervosa
		Psychogenic vomiting
		Eating disorders
F51	Sleep disorders	Nonorganic insomnia
		Nonorganic hypersomnia
		Sleepwalking
		Nightmares
		Sleep disorders
F04-F09、F52-F99	Other mental disorders	Organic mental disorder（except dementia）
		Sexual dysfunction, not caused by organic disorder or disease, uerperal mental disorder
		Personality disorders, habit and impulse disorders, gender identity disorders, disorders of sexual preference
		Mental retardation
		Disorders of psychological development
		Hyperkinetic disorders, conduct disorders, Emotional disorders with onset specific to childhood, Tic disorders
		Unspecified mental disorder
Not indicated	Undetermined diagnoses	Diagnosis coded F00-F99 to be investigated
		Symptomatic state of mental illness
		Psychotherapy

Table S2 Gender composition of age, diagnosis and year at the level of person-time of diagnosis and treatment

	Males		Females		Male-to-female ratio	*p*-values
	n	%	n	%		
Age group						< 0.001
0-18	8,478	36.39	14,818	63.61	1:1.75	
19-34	21,097	40.46	31,048	59.54	1:1.47	
35-49	22,528	41.44	31,829	58.56	1:1.41	
50-64	18,100	32.37	37,817	67.63	1:2.09	
≥65	10,153	33.63	20,040	66.37	1:1.97	
Diagnosis category						< 0.001
Dementia	148	40.66	216	59.34	1:1.46	
Mental and behavioral disorders due to psychoactive substance use	758	95.23	38	4.77	1:0.05	
Schizophrenia and other psychotic disorders	9,239	45.51	11,064	54.49	1:1.20	
Mood disorder	11,164	31.15	24,678	68.85	1:2.21	
Anxiety disorder	29,297	36.84	50,224	63.16	1:1.71	
Eating disorder	22	3.58	593	96.42	1:26.95	
Sleep disorder	11,351	38.73	17,954	61.27	1:1.58	
Other mental disorders	6,866	43.44	8,938	56.56	1:1.30	
Undetermined diagnoses	11,511	34.51	21,847	65.49	1:1.90	
Year						< 0.001
2019	41,680	37.65	69,019	62.35	1:1.66	
2020	38,676	36.76	66,533	63.24	1:1.72	

Table S3 Gender composition of age, diagnosis and year at the level of patient number

	Males		Females		Male-to-female ratio	*p*-values
	n	%	n	%		
Age group						< 0.001
0-18	3,343	38.07	5,438	61.93	1:1.63	
19-34	7,339	38.37	11,790	61.63	1:1.61	
35-49	6,475	40.07	9,685	59.93	1:1.50	
50-64	5,162	31.53	11,210	68.47	1:2.17	
≥65	2,918	35.12	5,390	64.88	1:1.85	
Diagnosis category						< 0.001
Dementia	62	35.23	114	64.77	1:1.84	
Mental and behavioral disorders due to psychoactive substance use	205	94.91	11	5.09	1:0.05	
Schizophrenia and other psychotic disorders	1,822	44.17	2,303	55.83	1:1.26	
Mood disorder	2,751	31.16	6,079	68.84	1:2.21	
Anxiety disorder	7,710	36.09	13,651	63.91	1:1.77	
Eating disorder	11	5.47	190	94.53	1:17.27	
Sleep disorder	4,711	37.99	7,690	62.01	1:1.63	
Other mental disorders	2,299	44.58	2,858	55.42	1:1.24	
Undetermined diagnoses	5,666	34.80	10,617	65.20	1:1.87	
Year						0.003
2019	12,538	37.26	21,109	62.74	1:1.68	
2020	12,699	36.18	22,404	63.82	1:1.76	

Table S4 Gender composition of age, diagnosis and year at the level of first-visit patient number

	Males		Females		Male-to-female ratio	*p*-values
	n	%	n	%		
Age group						< 0.001
0-18	2,753	38.46	4,405	61.54	1:1.60	
19-34	5,012	36.28	8,801	63.72	1:1.76	
35-49	3,575	39.15	5,556	60.85	1:1.55	
50-64	2,685	30.42	6,142	69.58	1:2.29	
≥65	1,546	36.35	2,707	63.65	1:1.75	
Diagnosis category						< 0.001
Dementia	46	34.33	88	65.67	1:1.91	
Mental and behavioral disorders due to psychoactive substance use	144	96.00	6	4.00	1:0.04	
Schizophrenia and other psychotic disorders	444	43.11	586	56.89	1:1.32	
Mood disorder	1,216	29.80	2,865	70.20	1:2.36	
Anxiety disorder	3,749	34.50	7,118	65.50	1:1.90	
Eating disorder	5	3.50	138	96.50	1:27.60	
Sleep disorder	3,385	37.39	5,668	62.61	1:1.67	
Other mental disorders	1,714	45.83	2,026	54.17	1:1.18	
Undetermined diagnoses	4,868	34.81	9,116	65.19	1:1.87	
Year						< 0.001
2019	8,097	36.87	13,865	63.13	1:1.71	
2020	7,474	35.22	13,746	64.78	1:1.84	

Table S5 Gender difference by age groups in 2019 and 2020.

	2019		2020		χ^2	*p*-values
	Males (%)	Females (%)	Males (%)	Females (%)		
Total patient visits level						
Total	37.65	62.35	36.76	63.24	18.308	< 0.001
0-18	37.50	62.50	35.53	64.47	9.611	0.002
19-34	42.28	57.72	38.64	61.36	71.657	< 0.001
35-49	40.92	59.08	42.06	57.94	7.257	0.007
50-64	32.67	67.33	32.03	67.97	2.649	0.104
≥65	33.28	66.72	34.00	66.00	1.746	0.186
Patient number level						
Total	37.26	62.74	36.18	63.82	8.736	0.003
0-18	39.36	60.64	37.05	62.95	4.896	0.027
19-34	39.73	60.27	37.11	62.89	13.895	< 0.001
35-49	40.13	59.87	40.01	59.99	0.025	0.874
50-64	31.61	68.39	31.45	68.55	0.050	0.824
≥65	35.32	64.68	34.94	65.06	0.129	0.720
First-visit patient number level						
Total	36.87	63.13	35.22	64.78	12.693	< 0.001
0-18	39.57	60.43	37.53	62.47	3.129	0.077
19-34	37.81	62.19	34.80	65.20	13.603	< 0.001
35-49	39.70	60.30	38.50	61.50	1.355	0.244
50-64	31.01	68.99	29.73	70.27	1.721	0.190
≥65	36.16	63.84	36.55	63.45	0.068	0.794

Table S6 Gender difference by diagnositic categories in 2019 and 2020

	2019		2020		χ^2	*p*-values
	Males (%)	Females (%)	Males (%)	Females (%)		
Total patient visits level						
Total	37.65	62.35	36.76	63.24	18.308	< 0.001
Dementia	39.63	60.37	42.18	57.82	0.235	0.628
Mental and behavioral disorders due to psychoactive substance use	94.23	5.77	96.42	3.58	2.088	0.148
Schizophrenia and other psychotic disorders	45.36	54.64	45.68	54.32	0.201	0.654
Mood disorder	32.25	67.75	29.76	70.24	25.630	< 0.001
Anxiety disorder	37.35	62.65	36.28	63.72	9.781	0.002
Eating disorder	5.30	94.70	2.11	97.89	4.513	0.034
Sleep disorder	38.35	61.65	39.11	60.89	1.802	0.179
Other mental disorders	41.98	58.02	44.76	55.24	12.462	< 0.001
Undetermined diagnoses	36.02	63.98	33.30	66.70	27.142	< 0.001
Patient number level						
Total	37.26	62.74	36.18	63.82	8.736	0.003
Dementia	33.00	67.00	38.16	61.84	0.503	0.478
Mental and behavioral disorders due to psychoactive substance use	94.59	5.41	95.24	4.76	0.046	0.830
Schizophrenia and other psychotic disorders	44.10	55.90	44.24	55.76	0.008	0.929
Mood disorder	31.60	68.40	30.69	69.31	0.850	0.357
Anxiety disorder	37.02	62.98	35.19	64.81	7.801	0.005
Eating disorder	6.67	93.33	4.50	95.50	0.449	0.503
Sleep disorder	37.78	62.22	38.19	61.81	0.225	0.635
Other mental disorders	43.95	56.05	45.13	54.87	0.723	0.395
Undetermined diagnoses	36.11	63.89	33.63	66.37	11.041	0.001
First-visit patient number level						
Total	36.87	63.13	35.22	64.78	12.693	< 0.001
Dementia	32.00	68.00	37.29	62.71	0.410	0.522

	2019		2020			
	Males (%)	Females (%)	Males (%)	Females (%)	χ^2	*p*-values
Mental and behavioral disorders due to psychoactive substance use	96.30	3.70	95.65	4.35	0.040	0.841
Schizophrenia and other psychotic disorders	43.82	56.18	42.14	57.86	0.291	0.590
Mood disorder	30.12	69.88	29.39	70.61	0.262	0.609
Anxiety disorder	36.23	63.77	32.62	67.38	15.655	< 0.001
Eating disorder	4.62	95.38	2.56	97.44	0.442	0.506
Sleep disorder	37.40	62.60	37.38	62.62	0.000	0.983
Other mental disorders	45.49	54.51	46.16	53.84	0.168	0.682
Undetermined diagnoses	36.02	63.98	33.67	66.33	8.458	0.004
